# Conversational AI in Cognitive and Social Training for People with Dementia: A Systematic Review

**DOI:** 10.3390/healthcare14142106

**Published:** 2026-07-14

**Authors:** Mark K. K. Chan, Peter H. F. Ng, Karen P. Y. Liu

**Affiliations:** Department of Rehabilitation Sciences, The Hong Kong Polytechnic University, Hong Kong SAR, China; peter.nhf@polyu.edu.hk (P.H.F.N.); karen.liu@polyu.edu.hk (K.P.Y.L.)

**Keywords:** conversational AI, dementia, chatbot, socially assistive robot, cognitive training, systematic review, large language model, mild cognitive impairment

## Abstract

Background: Conversational artificial intelligence (AI), including text-based chatbots, voice-based agents, multimodal systems, and socially assistive robots (SARs), offers a scalable adjunct to therapist-led dementia care. The post-2022 emergence of large language models (LLMs) has accelerated development, yet few reviews apply a unified conversational AI taxonomy across dementia care. This review synthesized the effectiveness, limitations, and implementation challenges of conversational AI across the dementia care continuum. Methods: Six databases (PubMed, Embase, Web of Science, Scopus, IEEE Xplore, ACM Digital Library) were searched for English-language studies (January 2010–March 2026) evaluating conversational AI targeting cognitive, social, or caregiver outcomes. Two reviewers independently screened and extracted data following PRISMA 2020 guidelines; risk of bias used standard tools and findings were synthesized narratively. Protocol: PROSPERO CRD420261333625. Results: Forty studies (8 randomized controlled trials [RCTs], 32 non-randomized) were included. SARs were the largest category (*n* = 24; 60.0%), followed by text-based chatbots (*n* = 12; 30.0%), multimodal systems (*n* = 3; 7.5%), and voice-based chatbots (*n* = 1; 2.5%). The strongest cognitive evidence came from a social robot RCT (gain of 3.9 points on a 30-point screening measure (*p* < 0.001). For caregivers, an international RCT (*n* = 274) showed significant reductions in depression (*d* = 0.37) and burden (*d* = 0.34). LLM-based systems produced an 18-fold increase in conversation duration. Speech recognition failure was the most consistently reported technical barrier. Conclusions: Conversational AI shows directional benefit across cognitive, social, and caregiver outcomes. Critical research gaps remain regarding voice-only randomized evidence and adequately powered LLM trials against usual care.

## 1. Introduction

Dementia represents one of the most pressing public health challenges of the twenty-first century. The global prevalence is projected to triple from 57 million in 2019 to 153 million by 2050, driven by population ageing [1]. In China alone, epidemiologic studies have documented a substantial and geographically variable dementia burden, and national estimates suggest that approximately 15 million individuals are affected, representing more than a quarter of the global burden [2,3,4,5].

The condition is characterized by a progressive decline in cognitive function and loss of independence in activities of daily living [6,7]. Dementia is frequently accompanied by behavioral and psychological symptoms (BPSD) which include depression, anxiety, and agitation which substantially diminish quality of life and increase caregiver burden [8,9]. The total economic cost in China alone is projected to reach 507.4 billion RMB by 2030, underscoring the urgent need to develop effective interventions that support cognitive function and social engagement [10].

Recent advances in artificial intelligence have positioned conversational agents, or chatbots, as promising digital health tools [11,12]. These systems simulate human dialogue through natural language processing (NLP) and machine learning, offering distinctive advantages for dementia care: 24/7 availability, personalized content delivery, a non-judgmental interaction environment, and the capacity for continuous data collection to inform clinical practice [13].

Preliminary evidence suggests that chatbots can enhance cognitive and social outcomes. For example, the Computer Interactive Reminiscence and Conversation Aid (CIRCA) improved social interaction quality through multimedia-facilitated reminiscence [14]. Other interventions have demonstrated improvements in short-term memory [15] and reductions in loneliness and depressive symptoms [16]. Related digital dementia-care interventions have also reported improved assisted living outcomes and promising automated speech-based assessment capabilities, although the evidence base remains methodologically heterogeneous [17,18]. However, the existing literature is constrained by small sample sizes, limited long-term follow-up, and a primary focus on mild cognitive impairment (MCI) or early-stage dementia. Technical challenges particularly in processing impaired speech and ensuring data security also persist.

Related artificial intelligence (AI) work in dementia care spans explainable detection and prognosis [19] and DSM-5-based monitoring assistants [20], though these address detection and monitoring rather than the conversational interventions synthesized here. Given the growing interest in this domain, a systematic evaluation of conversational AI applications for dementia care is warranted. This review aims to synthesize current research on conversational AI interventions, encompassing text-based chatbots, voice-based agents, multimodal systems, and socially assistive robots, to assess their effectiveness across cognitive, social, and caregiver outcomes, delineate existing limitations, and propose directions for future research to guide clinicians, researchers, and technology developers.

## 2. Materials and Methods

We conducted this systematic review in accordance with the Preferred Reporting Items for Systematic Reviews and Meta-Analyses (PRISMA) 2020 guidelines [21,22]. The completed PRISMA checklist is included as Supplementary File S1. The review protocol was prospectively registered on the International Prospective Register of Systematic Reviews (PROSPERO); registration number: CRD420261333625.

### 2.1. Search Strategy

#### 2.1.1. Study Identification

A broad literature search was performed across six electronic databases, PubMed, Embase, Web of Science, Scopus, IEEE Xplore, and the ACM Digital Library. It covered the studies from 1 January 2010 to 31 March 2026. Search terms were structured around three core concepts: (1) Conversational AI and Technology; (2) the targeted population; and (3) the application domain. Boolean operators were applied to combine these terms, ensuring a broad yet relevant retrieval of the literature. Table 1 showed the search string used across databases. The final search was executed across all six databases on 26 June 2026, and the full search strategy is available in Supplementary File S2.

The search period was delimited to January 2010 onwards to coincide with the onset of the modern AI era in healthcare. The year 2010 marked a pivotal transition: the convergence of increased computational power, large-scale digitized health data, and the maturation of machine learning, particularly deep learning which enabled the development of NLP systems capable of supporting genuine human–computer dialogue [13]. Prior to this period, conversational systems in healthcare were largely confined to rule-based expert systems with limited interactivity and minimal clinical application data, rendering earlier literature of marginal relevance to the current review scope. The rationale for the upper boundary of March 2026 was to capture the most current evidence, particularly given the accelerating pace of LLM-driven development. The marked surge in publications after 2023 reflects the transformative impact of accessible large language models: the public release of ChatGPT (GPT-3.5) in November 2022 and GPT-4 in March 2023 substantially lowered the technical barrier to building sophisticated conversational AI applications, prompting a rapid expansion of research into their clinical and therapeutic potential in dementia care [11].

#### 2.1.2. Inclusion and Exclusion Criteria

Inclusion and exclusion criteria were developed a priori following the PICOS framework [21,22] and prospectively registered on PROSPERO (CRD420261333625); they are summarized in Table 2. Studies were eligible if they were (i) published in English between January 2010 and March 2026 in peer-reviewed journals or formal conference proceedings, (ii) reported original empirical data on a conversational AI intervention as defined in Table 3, and (iii) targeted persons with dementia (PwD), persons with mild cognitive impairment (MCI), or their formal or informal caregivers, dementia population which account for equal to or more than half in mixed population are included. We excluded narrative and scoping reviews, editorials and commentaries, conference abstracts without sufficient data for extraction, and animal studies. We further excluded studies in which (a) the comparator was another conversational AI, large language model–based chatbot, or advanced spoken dialogue system, because the review aims to isolate the effect of conversational AI against non-AI or standard care; (b) the conversational agent was embedded within a complex multi-component intervention such that AI-specific effects could not be isolated; (c) only pharmacological comparators were employed; or (d) the focus was purely on technical or algorithmic development of NLP systems without participant interaction or user evaluation data. Studies in severe or end-stage dementia were eligible where the intervention was directed at the caregiver or at preserved social-engagement capacities. Programs lacking interactive conversational exchange (e.g., one-way e-learning or static web-based information programs) were excluded at screening under the non-conversational digital tools criterion.

#### 2.1.3. Study Selection and Data Extraction

The study selection process was managed using EndNote 2025 for citation management and duplicate removal. Two reviewers (K.K.C. & P.N.) independently screened titles and abstracts, followed by full-text review of potentially eligible articles, adhering to the PRISMA 2020 guidelines. Inter-rater agreement was assessed using Cohen’s kappa [25]; agreement was substantial for both title/abstract screening and full-text review. Discrepancies were resolved through discussion. If consensus could not be reached, a third reviewer (an occupational therapist with expertise in dementia care) was consulted (K.L.). The review protocol was registered in PROSPERO. A structured data extraction form (Table 4), developed in consultation with clinical and technical experts, covered: study characteristics (authors, year, country), participant details (sample size, age, dementia severity), chatbot system features (type, AI technology, interaction modality), application context (cognitive or social training), primary outcomes, and reported challenges or limitations.

### 2.2. Data Synthesis

Owing to the heterogeneity of interventions, study designs, and outcome measures across the included articles, a quantitative meta-analysis was not feasible. A narrative synthesis approach was, therefore, employed, providing a comprehensive summary of technological characteristics, application effectiveness, and implementation challenges. This method facilitated the identification of developmental patterns, evaluation of impacts on cognitive and social outcomes, and exploration of future research trajectories from both clinical and human–computer interaction perspectives. The review was conducted in line with the registered protocol (PROSPERO CRD420261333625). One amendment was logged: the search strategy was revised to improve sensitivity, with a corresponding extension of the review timeline. In deviation from the protocol’s planned quantitative synthesis, no meta-analysis was performed owing to substantial clinical and methodological heterogeneity, and a narrative synthesis was adopted. Consistent with the protocol, reporting bias and certainty of evidence (GRADE) were not formally assessed.

### 2.3. Risk of Bias Assessment

Risk of bias for RCTs was assessed using the revised Cochrane Risk of Bias Tool version 2.0 (RoB 2) [66], which evaluates five domains: (D1) bias from the randomization process, examining sequence generation and allocation concealment; (D2) bias due to deviations from intended interventions, including the effectiveness of blinding; (D3) bias due to missing outcome data; (D4) bias in measurement of the outcome, including assessor blinding; and (D5) bias in selection of the reported result. For each domain, a judgment of “low risk,” “some concerns,” or “high risk” was assigned; an overall rating of high risk was applied if any domain was high risk, or if two or more domains were rated some concerns. Non-randomized studies were assessed using using the Risk of Bias in Non-Randomized Studies of Interventions (ROBINS-I) tool (2016 version)z-[67], which evaluates seven domains (D1a–D7), with ratings of “low,” “moderate,” “serious,” or “critical” risk. All assessments were completed in duplicate; disagreements were resolved by discussion.

### 2.4. Outcome Measures

Outcomes were pre-specified in the PROSPERO protocol (CRD420261333625) across four domains: (1) cognitive function and performance (e.g., MMSE, MoCA, ADAS-cog, fNIRS); (2) social engagement and emotional well-being (e.g., GDS, NPI, UCLA Loneliness Scale); (3) caregiver burden and mental health (e.g., ZBI, PHQ-9); and (4) system acceptability, adherence, and technical feasibility (e.g., SUS, MAUQ, word error rate). All reported measurement timepoints were eligible.

## 3. Results

The results are organized into thematic sections aligned with the systematic review’s research questions, encompassing study selection and characteristics of the included evidence, intervention type and technological classification, risk of bias, and the effects of conversational AI on cognitive function and performance, social engagement and emotional well-being, caregiver burden and mental health, and system acceptability, adherence, and technical feasibility.

### 3.1. Study Selection

**Figure 1:**
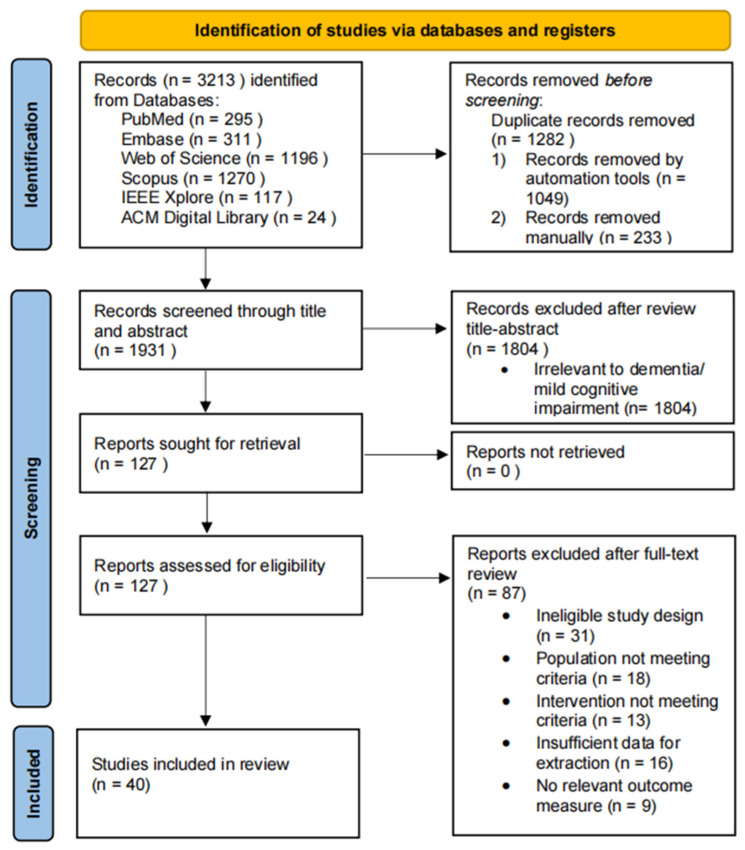
PRISMA 2020 flow diagram illustrating study identification, screening, and inclusion. Colours indicate PRISMA 2020 phases: orange = identification of studies via databases and registers; blue = review stages (identification, screening, included).

A systematic search of six databases (PubMed, Embase, Web of Science, SCOPUS, IEEE Xplore, ACM Digital Library) identified 3213 records. Following removal of 1282 duplicates via automated tools (*n* = 1049) and manual review (*n* = 233), 1931 records proceeded to title/abstract screening; 1804 studies were excluded due to the reason of irrelevance to dementia or mild cognitive impaired population. All 127 full-text reports were retrieved; 87 were excluded (ineligible design *n* = 31; ineligible intervention *n* = 13; ineligible population *n* = 18; insufficient data *n* = 16; irrelevant outcome measure *n* = 9). Forty studies were included in the final synthesis. Full PRISMA 2020 flow diagram is presented in Figure 1.

### 3.2. Study Characteristics

**Figure 2:**
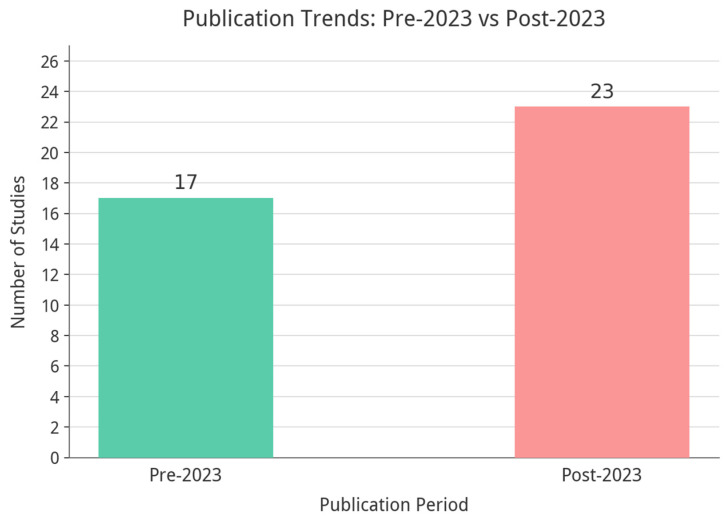
Publication trends of included studies: pre-2023 versus 2023 onwards.

The forty included studies were published between 2015 and 2026, with marked acceleration from 2023 onwards (Figure 2). Pre-2023 publications (*n* = 17) [27,32,34,35,36,37,42,43,44,45,47,48,49,58,61,62,63] accumulated steadily across seven years, whereas studies from 2023 onwards (*n* = 23) [26,28,29,30,31,33,38,39,40,41,46,50,51,52,53,54,55,56,57,59,60,64,65] comprise more than half the corpus within three years, which align with the field-wide inflection precipitated by accessible LLMs in late 2022 and 2023.

Studies were conducted across sixteen single-country settings spanning five continents, plus one multinational program conducted across 43 countries [26]. The corpus was concentrated in North America (*n* = 8), Southern and Western Europe (*n* = 12), and East Asia (*n* = 12), with smaller contributions from Mexico, Peru (in collaboration with the United Kingdom), and Australia. Africa and South Asia were unrepresented despite the asymmetry of the global dementia epidemic.

Designs included eight randomized controlled trials, five full RCTs [26,27,28,29,30] and three pilot RCTs [31,32,33]. Thirty-two non-randomized studies [34,35,36,37,38,39,40,41,42,43,44,45,46,47,48,49,50,51,52,53,54,55,56,57,58,59,60,61,62,63,64,65] including pilot and feasibility studies, quasi-experimental and cross-sectional comparative designs, mixed-methods evaluations, observational studies, and design or usability evaluations.

Sample sizes ranged from *n* = 4 [62] to *n* = 274 [26]; most non-randomized studies recruited fewer than thirty participants. Participants included persons with dementia of varying severity, MCI, and Alzheimer’s disease, alongside informal and formal caregivers. Settings spanned community-dwelling, nursing and residential care, day care centers, and outpatient memory clinics. Intervention durations ranged from two weeks to approximately six months.

### 3.3. Intervention Types

The forty studies were classified by primary interaction modality (Table 5): socially assistive robots (SARs; *n* = 24; 60.0%), text-based chatbots (*n* = 12; 30.0%), multimodal systems (*n* = 3; 7.5%), and voice-based chatbots (*n* = 1; 2.5%). SARs encompassed humanoid platforms (Pepper, MARIO, NAO, Kabochan, PIO, RoBoHoN, Eva). Text-based chatbots were primarily caregiver-facing platforms delivered via web or smartphone (PDC30, CareBuddy, CareHeroes, Ana, ADQueryAid). The single voice-only intervention was a telephone-delivered service (CLOVA CareCall; [65]), it indicated a striking evidence gap given the modality’s low scaling barrier, particularly in resource-limited settings.

A secondary classification by technological stratum revealed a sharp generational shift. Approximately one-fifth of studies (*n* = 8; 20%) deployed LLM-based or generative AI systems, exclusively published in 2024–2026 and post-dating the public release of GPT-4 in March 2023. These spanned GPT-3.5–powered [41,53],GPT-4 and GPT-4o–powered [40,56], retrieval-augmented generation systems [50], and personality-adaptive or embodied agents [55,59]. The remaining studies employed rule-based dialogue systems or traditional NLP pipelines.

### 3.4. Risk of Biases

**Figure 3:**
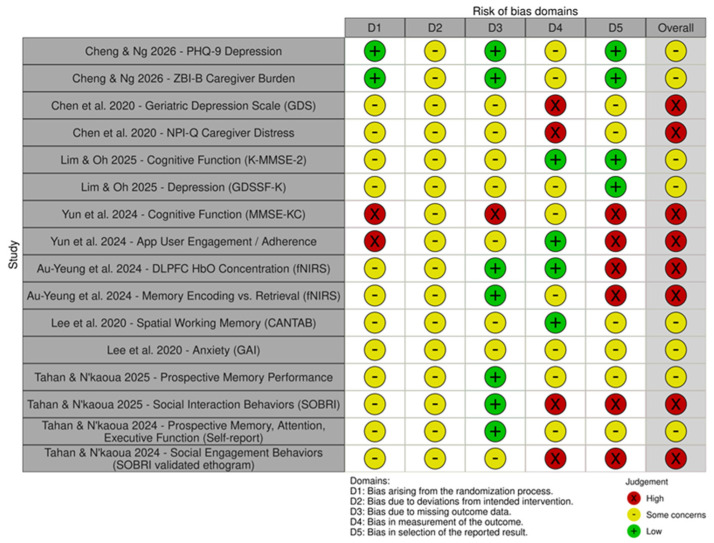
Summary of risk of bias assessment (RoB 2) [26,27,28,29,30,31,32,33].

**Figure 4:**
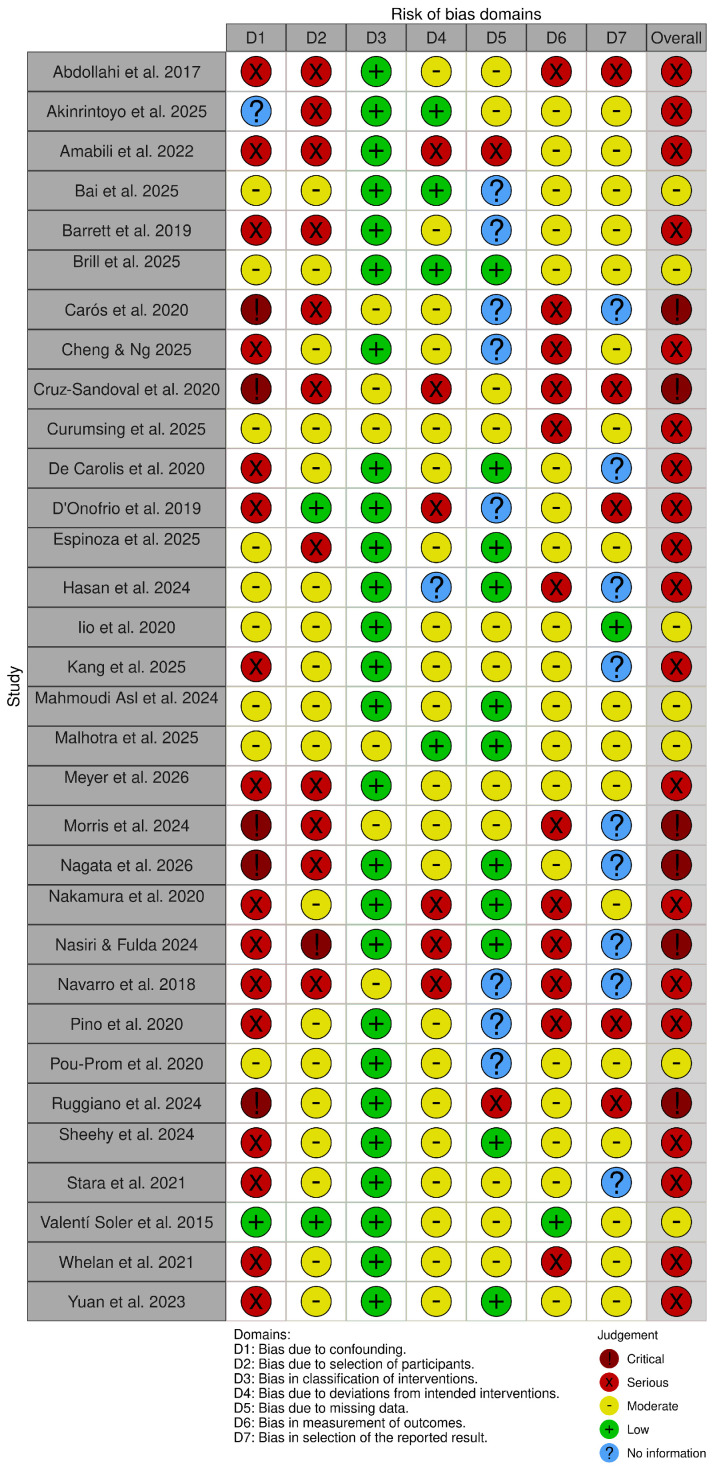
Summary of risk of bias assessment (ROBINS-I) [34,35,36,37,38,39,40,41,42,43,44,45,46,47,48,49,50,51,52,53,54,55,56,57,58,59,60,61,62,63,64,65].

Risk of bias was assessed at the outcome level for randomized trials using RoB 2 and at the study level for non-randomized studies using ROBINS-I (Figure 3 and Figure 4). Across eight RCTs (sixteen outcome assessments), three trials had all outcomes rated “Some concerns” [26,28,32], reflecting concerns about participant blinding inherent to visible AI interventions. Five trials yielded one or more “High risk” outcomes [27,29,30,31,33], driven predominantly by selective outcome reporting (D5) and outcome measurement (D4).

Among thirty-two non-randomized studies assessed via ROBINS-I, seven were rated “Moderate” [39,45,47,49,50,54,57] while nineteen were rated “Serious” [34,35,36,37,38,40,41,42,43,44,48,52,53,56,58,59,61,64,65], predominantly reflecting uncontrolled confounding, convenience sampling, and selective reporting. Six studies were rated “Critical” [46,51,55,60,62,63]. The aggregate distribution (Moderate 22%, Serious 59%, Critical 19%) is characteristic of an emerging digital health field. Findings derived from Serious or Critical evidence should be interpreted as directional rather than confirmatory.

### 3.5. Cognitive Function and Performance

Cognitive benefit was directionally consistent across studies but reached statistical significance in only a minority of adequately controlled trials, with effect sizes ranging from clinically meaningful (K-MMSE-2 gain 3.9 points) to null (equivalence with usual care). Five randomized trials supported the cognitive evidence. The PIO social robot produced a clinically meaningful K-MMSE-2 gain in mild-to-moderate dementia (3.9 ± 3.66 vs. minimal change in controls; *t* = 3.94, *p* < 0.001; *n* = 66) [28]. An AI chatbot mHealth application in rural older adults at risk of dementia produced significant cognitive improvement with a dose–response between usage and effect (*n* = 123) [30]. Spatial working memory improved more in MCI participants randomized to a home-based personal robot than to control [32]. Two further RCTs yielded equivalence rather than superiority: NAO-delivered prospective memory training matched usual care on cognition [33] and DLPFC haemodynamic activation on fNIRS was comparable between robot-led and human-led training [31].

Voice-based evidence is sparse but directionally positive: a telephone-delivered service produced significant memory improvement (*n* = 80) with no change in attention or language [65]. Non-randomized work documented gains in prose memory and verbal fluency with NAO-mediated training in MCI [34] and linguistic features extracted from robot-led conversations have been proposed as cognitive monitoring signals [45].

### 3.6. Social Engagement and Emotional Well-Being

Evidence in this domain concerns predominantly PwD in residential and community settings, with limited MCI-specific data. Three randomized trials anchored social engagement and emotional well-being. An ABAB withdrawal RCT of the Kabochan companion robot in nursing-home residents (*n* = 103) recorded significant reduction in neuropsychiatric-related caregiver distress at week 16 *p* = 0.011), with symptom re-emergence on withdrawal at week 24 (*p* = 0.003), a pattern associated with a causal contribution [27]. Telephone-delivered AI produced significant GDS depression reduction [65], while non-significant between-group depression change in the PIO arm suggests cognitive benefits of robot-mediated programs do not necessarily translate to mood.

In non-randomized studies, sustained engagement with the Ryan Companionbot was recorded over 4–6 weeks (mean 198 dialogues/day) without observable decay [42], and significant resilience improvement (RS-14: *p* = 0.020) followed MARIO interaction with age-stratified gains in depression and perceived social support [36,61].

BPSD effects were heterogeneous: significant NPI-NH reduction followed Eva-facilitated cognitive stimulation therapy [63], and phase-dependent apathy improvements with NAO were reported [47]. The UCLA Loneliness which used to measure loneliness was rarely employed across included studies, representing a substantive measurement gap.

### 3.7. Caregiver Burden and Mental Health

This domain concerns informal and formal caregivers rather than PwD or MCI participants directly. Caregiver-focused interventions, delivered predominantly through text-based chatbots, produced the most methodologically rigorous evidence. The only adequately powered international waitlist-controlled RCT (*n* = 274 caregivers across 43 countries) reported significant 1-month improvements in caregiver depression (*d* = 0.37), burden (*d* = 0.34), and positive gains (*d* = 0.42) following the PDC30 automated psychoeducation platform [26]. Companion-robot exposure also significantly reduced NPI-Q caregiver distress [27].

Pilot evidence is convergent. A CareHeroes app and AI chatbot pilot (*n* = 21) recorded significant reduction in caregiver depression at 3-month follow-up (t11 = 2.03, *p* = 0.03) [46]. PDC30 chatbot acceptability was confirmed in a Hong Kong sample (*n* = 21) with daily-plus engagement over two weeks [56]. Caregivers in Peru significantly preferred a generative AI Ana chatbot (Ana-V2) over a rule-based version on empathy, understanding, and knowledgeability (Wilcoxon, *p* < 0.05) [53]; in Taiwan, formal caregivers preferred a screen-equipped social robot to an equivalent tablet on UEQ, TAM, and perceived supportiveness [57].

Caregiver-facing LLM systems are reaching technically respectable performance but with proxy-population evaluation. A personality-aware chatbot tested in students (*n* = 24) found that 70% reported the enhanced model “got to know them better,” with conversation length increasing by 12.25% [55]. A case study of the Ted embodied conversational agent (*n* = 23 caregivers) demonstrated retention of learned communication principles at eight weeks alongside an emotional connection with the agent [59].

### 3.8. System Acceptability, Adherence, and Technical Feasibility

PwD and caregivers who used the conversational AI broadly accept the systems when delivered well, but speech recognition remains the dominant technical barrier. SUS scores at or above the conventional 68 benchmark were reported across multiple platforms: SUS 65.4→73.8 with MAUQ 95.4 [50] SUS 68.3 [54]; and 81% adherence to agent-initiated turns [39]. All Moderate RoB. PwD also enjoyed and were willing to adopt a socially assistive system long-term [52].

Speech recognition was the most consistently reported technical limitation, modality independent. A word error rate of 0.778 in a twin-robot dialogue system still produced mean dialogue durations of 12 min 51 s, it indicating tolerance of substantial recognition error when conversational structure is preserved [49]. Speech recognition failures reduced intention to use, perceived usefulness, and trust, while a touchscreen modality was positively used by PwD. The cleanest evidence that multimodal fallback materially mitigates speech-recognition limitations [58]. Voice control was “largely unsuccessful” in another platform although non-verbal interaction continued [38]; recognition failure was particularly pronounced in mild dementia [54].

LLM integration manifested most strikingly in conversation depth. A GPT-3.5 upgrade in long-term care residents produced an 18-fold increase in mean session length (2:48 → 53:50 min), with six of ten participants requesting further interaction and no simulator sickness reported [41]. Conversation length increased by 12.25% with personality-aware modules [55], and caregivers significantly preferred a generative chatbot over a rule-based version despite awareness of inaccuracy risks [53]. Randomized efficacy data against usual care remain absent for LLM-based systems. These LLM-related observations, specifically the 18-fold increase in conversation duration and the preference for generative over rule-based systems, demonstrate increased engagement and conversational depth rather than improved cognitive, behavioral, or caregiver outcomes; in the absence of adequately powered trials, therapeutic superiority cannot be inferred from conversational depth alone.

Adherence followed engagement quality. Dose–response was documented between application use frequency and cognitive improvement [30]; 8-week retention of caregiver learning was found [59]; and undiminished interest was recorded across 4–6 weeks [42]. In a Latino caregiver feasibility study (*n* = 55; Serious RoB), 50.9% of caregivers registered for the NeuViCare app, and registrants attended significantly more group sessions than non-users (2.9 vs. 2.0 of 4; *p* = 0.002), although interactive AI features were used infrequently. It suggested adoption of conversational features lags adoption of platform infrastructure [40].

## 4. Discussion

### 4.1. Principal Findings

This review synthesized 40 empirical studies evaluating conversational AI in cognitive and social training for PwD or MCI and their caregivers. It is the first systematic synthesis to apply a conversational AI taxonomy across this care continuum and to capture the post-GPT-4 wave of LLM-based applications. These findings should be read as directional rather than confirmatory: only 8 of 40 studies were randomized, no RCT outcome achieved an overall low risk of bias, and most non-randomized studies carried moderate-to-critical risk of bias. Because the strongest reported effects derive disproportionately from small, non-randomized, or higher-risk studies, their reliability is correspondingly uncertain. Four principal findings emerge, which together reconfigure the practical priorities for digital dementia care: the strongest controlled evidence sits in the lowest-cost modalities; the LLM transition has measurably changed interaction depth; socially assistive robots remain the broadest but not the strongest evidence base; and speech recognition, not algorithmic sophistication remains the binding technical constraint.

First, the strongest controlled evidence is for caregiver outcomes [26] and cognitive function in PwD [28,30] derives from low-tech delivery modalities (web platforms, smartphone applications, telephone services), but not from the more visible socially assistive robotics that dominate by study count. The single voice-only telephone study produced a clear depression-reduction signal at modest cost [65]. Scalability and clinical effect appear partially decoupled from technological sophistication.

Second, the LLM transition is empirically measurable. An 18-fold conversation length increase followed a GPT-3.5 upgrade [41]; significant user preference for generative over rule-based chatbots on empathy and understanding was demonstrated [53]; and 8-week retention of an emotional connection to an LLM-based embodied agent was recorded [59]. However, no LLM-based system in this corpus has yet been evaluated in an adequately powered RCT against usual care, and clinical-accuracy and safety questions remain open: caregiver-targeted LLM systems with promising backend performance [55] have been evaluated primarily against proxy populations. Accordingly, these findings should be interpreted as evidence of engagement and acceptability, not of therapeutic efficacy.

Third, SARs remain the modality with the broadest evidence base (*n* = 24, including six RCTs) and strongest evidence for engagement and BPSD reduction, but with weaker direct cognitive evidence and the greatest cost and maintenance burden. Comparable DLPFC haemodynamic activation between robot-led and human-led cognitive training [31] provides preliminary neurophysiological support for therapeutic equivalence, although the High RoB warrants replication.

Fourth, speech recognition for dysarthric, paraphasic, or accented speech is the most consistently reported technical barrier [38,49,52,54,58]. Touchscreen fallback positively used by PwD when speech failed [58] is the cleanest evidence that multimodal redundancy matters more than improving automatic speech recognition in isolation.

### 4.2. Clinical Implications

From a clinical implementation perspective, conversational AI is most credibly deployed today as an adjunct to therapist-led cognitive and psychosocial intervention. The evidence base supports three immediate clinical uses. First, caregiver psychoeducation and self-efficacy support via text-based chatbots can be integrated into discharge planning and community follow-up pathways, where 24-h availability addresses a gap that scarce clinician time cannot fill [26,46]. Second, socially assistive robots can augment group activity programming in residential and day care settings, particularly for reminiscence, sensory engagement, and BPSD-responsive companionship, where therapist attention must be distributed across many residents [27,36,63]. Third, voice-based telephone interventions warrant consideration as a low-cost, low-barrier option for community-dwelling older adults with limited digital literacy or device access [65]. Across all three uses, clinicians and allied-health professionals are well positioned to lead implementation, for example, selecting appropriate clients, grading task difficulty, monitoring engagement and adverse responses, and integrating chatbot interaction within the broader care plan rather than treating it as a standalone intervention. The Stara et al. [58] finding that PwD readily use touchscreen fallback when speech recognition fails directly aligns with established principles of activity grading and multimodal compensation: clinicians should expect, plan for, and design around recognition failure rather than treat it as an exclusion criterion. Routine adoption for direct cognitive training in PwD remains premature pending adequately powered controlled validation.

Selection and grading of conversational AI interventions should be matched to dementia severity and functional profile, applying the same clinical reasoning used for any graded activity. For older adults with subjective cognitive decline or mild cognitive impairment, text-based chatbots delivered via smartphone or web platforms (e.g., PDC30, CareBuddy) are accessible, low-cost, and align with preserved literacy and digital skills [26,30,50]. For moderate dementia, where reading and sustained text engagement become unreliable, multimodal systems or socially assistive robots that combine voice, visual, and gestural input channels are more appropriate. Besides, touchscreen and tablet fallback should be planned for from the outset rather than treated as a workaround [58]. For advanced dementia, where direct cognitive training is unlikely to yield meaningful gains, caregiver-mediated approaches such as chatbot-based caregiver psychoeducation and support are likely the most clinically useful application.

Practical integration into existing services will require training of clinicians and allied-health professionals in three areas. First, embedding digital literacy assessments within routine clinical profiling. Second, developing core competencies in chatbot and SAR interaction modes sufficient to model and coach client usage. Third, identifying critical conversational AI failure modes such as speech recognition, LLM-generated hallucinations, or simulator sickness in VR-embedded systems that warrant immediate clinical intervention or withdrawal. Service-level integration also requires clear referral pathways, protocols for managing inaccurate AI-generated advice. Particularly, given the population’s acute vulnerability, data privacy and informed consent emerge as highly amplified ethical concerns when cloud-based LLM systems process conversations with patients with dementia. These implementation requirements are not yet addressed by the included literature and represent a meaningful gap between feasibility evidence and routine clinical deployment.

The integration of conversational AI into dementia care introduces a layered set of risks that the current evidence base only partially addresses, spanning five interacting domains: generative reliability, data privacy and security, informed consent and capacity, user safety and over-reliance, and technical interaction failure. First, generative systems are prone to hallucination and factual inaccuracy, which is a particular hazard when caregivers act on AI-generated guidance, yet caregivers may prefer generative systems even while aware of this risk [26]. Second, cloud-based processing of sensitive conversational and clinical data raises data-privacy and security concerns that on-device or privacy-preserving architectures were rarely evaluated to address [46]. Third, the population’s fluctuating capacity to consent amplifies standard ethical requirements, with consent routinely obtained from surrogates and the prospect of attachment or deception when users relate to a system as sentient [27]. Fourth, vulnerable users may over-trust or become dependent on AI companionship where human attention is scarce, underscoring the need to study dependency and maintain escalation pathways to human care [61]. Fifth, and most consistently, speech-recognition and interaction failures remain the binding technical constraint, best mitigated through multimodal redundancy rather than treated as exclusion criteria [49,58]. Taken together, these considerations warrant explicit governance and reporting standards before LLM-based conversational AI is deployed at scale; the specific risks, the research needed to characterize them, and the governance controls required are summarized in Table 6.

### 4.3. Limitations

This review has several limitations. First, substantial heterogeneity precluded quantitative meta-analysis; effect sizes were synthesized narratively. Second, the English-language restriction produced geographic concentration in East Asia, Europe, and North America with Africa unrepresented. The exclusion of grey literature and non-English publications may additionally have omitted relevant studies and could bias the synthesis toward published, positive-result, English-language findings. Third, only eight of forty studies were RCTs, and only Cheng and Ng [26] achieved a sample size adequate for definitive inference. Risk of bias was Moderate to Critical for most non-randomized studies; no included RCT outcome achieved Low overall risk. A substantial proportion of included studies (*n* = 26) enrolled fewer than 30 participants, frequently in single-site convenience samples; such small cohorts increase the risk of unstable estimates and limit generalizability, and reported effect sizes should be regarded as preliminary signals requiring confirmation in adequately powered trials. Findings should, therefore, be interpreted as directional rather than definitive. In line with the registered protocol, certainty of findings was not formally graded (GRADE was not applied) and reporting bias was not assessed, which representing known limitations given the field’s likely positive-result publication bias. Also, speech-recognition performance metrics were reported in only a minority of studies despite qualitative documentation of speech failures, limiting cross-platform comparability. Finally, the voice-based chatbot category contained only a single study (*n* = 1; 2.5%), which limits the generalizability of findings for this modality; however, this classification was pre-specified in the registered PROSPERO protocol and the scarcity itself constitutes a substantive evidence gap finding.

## 5. Conclusions

Conversational AI applied to cognitive and social training for people with dementia and their caregivers shows consistent directional benefit across cognitive function, social engagement, emotional well-being, caregiver burden, and system acceptability. The strongest controlled evidence is concentrated in low-cost text-based and telephone-delivered modalities; SARs provide the broadest evidence for engagement and BPSD reduction; LLM-based systems demonstrate qualitatively superior interaction depth but lack adequately powered controlled efficacy data. The two most actionable evidence gaps are the near-absence of voice-only randomized evidence (a single study) and the absence of adequately powered randomized trials of LLM-based systems against usual care. Priorities for future research include adequately powered, multi-site RCTs of voice and LLM modalities; standardized outcome reporting; benchmarking of automatic speech recognition on dysarthric speech; clinical-accuracy and safety reporting for LLM-based caregiver systems; and inclusion of underrepresented populations across Africa, South Asia, and broader Latin America.

## Figures and Tables

**Table 1:** Search string used across databases.

Block	Concept	Search Terms (Terms Within Each Block Combined with OR)
1	Conversational AI & Technology	“chatbot” OR “conversational agent” OR “dialogue system” OR “virtual assistant” OR “conversational AI” OR “social robot *” OR “embodied conversational agent *” OR “large language model *” OR “LLM *” OR “ChatGPT” OR “digital companion *” OR “voice assistant *”
AND		
2	Target Population	“dementia” OR “Alzheimer’s disease” OR “cognitive impairment” OR “MCI” OR “older adult*” OR “elder *” OR “aging” OR “ageing” OR “elderly” OR “cognitive decline” OR “memory loss”
AND		
3	Intervention & Outcome	“cognitive training” OR “social engagement” OR “intervention” OR “therapy” OR “reminiscence *” OR “cognitive stimulation” OR “caregiver *”

Note. * = truncation wildcard. Blocks combined with AND; terms within each block combined with OR. Applied to title, abstract, and keywords in PubMed, SCOPUS, Embase, IEEE Xplore, ACM Digital Library, and Web of Science.

**Table 2:** PICOS eligibility criteria.

Criterion	Component	Inclusion	Exclusion
**P**	Population	Adults with dementia (any type/severity) or MCI; formal and informal caregivers	Populations without dementia or MCI diagnosis
**I**	Intervention	Conversational AI (text-based, voice-based, multimodal, or socially assistive robot) for cognitive/social training, social engagement or caregiver support; January 2010–March 2026; English-language	Non-conversational digital tools; technical-only AI studies
**C**	Comparator	Usual/standard care; waitlist control; non-AI digital tools; human-delivered therapy (e.g., CST)	Pharmacological comparators
**O**	Outcomes	Cognitive function (MMSE, MoCA); social engagement and emotional well-being (Geriatric Depression Scale (GDS), UCLA Loneliness Scale (UCLA-LS); caregiver burden; system usability and acceptability	Technical performance metrics (e.g., ASR accuracy, WER) without clinical outcomes
**S**	Study Design	RCTs; non-randomized trials; quasi-experimental; pilot, feasibility, and observational studies	Reviews; editorials; conference abstracts; animal studies; grey literature
**Dates**	Publication period	January 2010–March 2026	Publications before January 2010
**Lang** **.**	Language	English	Non-English publications

Note. MCI = mild cognitive impairment; SAR = socially assistive robot; CST = cognitive stimulation therapy; MMSE = Mini-Mental State Examination; MoCA = Montreal Cognitive Assessment; GDS = Geriatric Depressi on Scale; UCLA-LS = UCLA Loneliness Scale; ASR = automatic speech recognition; WER = word error rate; RCT = randomized controlled trial. PROSPERO registration: CRD420261333625.

**Table 3:** Definitions of conversational AI and interaction modalities used in this review.

Term	Definition	Distinguishing Feature
**Conversational AI**	An umbrella term for any computer system that simulates human dialogue through natural language processing, encompassing rule-based, retrieval-based, and generative (LLM) architectures capable of sustaining multi-turn natural-language exchange [23].	Must support ≥1 turn of bidirectional natural-language interaction; excludes passive information displays and one-way notifications.
**Text-based chatbot**	A conversational agent that receives and delivers information exclusively through on-screen text via web, mobile application, or messaging platform.	No voice input/output or physical embodiment; interaction is typed and read.
**Voice-based chatbot**	A conversational agent that operates entirely through spoken dialogue, typically delivered via telephone or voice-only device.	Audio-only channel; no visual interface, screen, or physical form factor.
**Multimodal chatbot**	A conversational agent that combines two or more interaction channels (e.g., voice with visual avatar, VR environment, or touchscreen) without a physically embodied robot body.	Screen-based or virtual embodiment; integrates visual, verbal, and/or tactile input but lacks physical presence in the user’s environment.
**Socially assistive robot (SAR)**	A physically embodied robot designed to provide social interaction and assistance through speech, gesture, gaze, and/or touch, operating in the user’s physical environment [24].	Physical presence distinguishes SARs from screen-based systems; interaction leverages embodiment cues (gesture, proximity, gaze direction).

Note. LLM = large language model; VR = virtual reality; SAR = socially assistive robot. Categories are mutually exclusive; each study was classified by its primary interaction channel as registered in PROSPERO (CRD420261333625). Systems combining multiple interaction modes were assigned to their primary channel; physically embodied systems were coded as socially assistive robots even when LLM-powered, and the use of LLM versus rule-based architecture was recorded separately under the technological-stratum classification (Section 3.3) rather than as a modality.

**Table 4:** Full data extraction information.

#	Authors (Year)	Country	Study Design	Sample/Population (N)	Intervention/System Used	Key Clinical Findings
1	Cheng and Ng (2026) [26]	International (43 countries)	Parallel-group randomized waitlist-controlled trial	Adults providing care to relatives with dementia; *n* = 274	PDC30; AUTOMATED	After one month, caregivers using PDC30 showed significant improvements in depression (*d* = −0.37), burden (*d* = −0.34), and positive gains (*d* = 0.42) compared to the waitlist control group. These differences largely disappeared once the control group received the intervention, with improvements sustained thereafter. The automated, self-guided intervention demonstrated viability and potential global health impact.
2	Chen et al. (2020) [27]	Hong Kong	RCT with ABAB withdrawal design	Residents with dementia; *n* = 103	Kabochan (humanoid companion robot); AUTOMATED	The Kabochan robot was associated with a statistically significant reduction in neuropsychiatric-related caregiver distress at week 16 (*p* = 0.011). When the robot was removed, neuropsychiatric symptoms became more severe in the intervention group at week 24 (*p* = 0.003). No significant differences were found in other health outcomes.
3	Lim & Oh (2025) [28]	South Korea	RCT	Older adults with mild to moderate dementia; *n* = 66	Social robot PIO; AUTOMATED	The social robot PIO program significantly improved cognitive function in older adults with mild to moderate dementia (t = 3.94, *p* < 0.001). While depression decreased in the experimental group compared to the control group, this difference was not statistically significant (z = −0.59, *p* = 0.557), suggesting a need for further research.
4	Tahan & N’kaoua (2023) [29]	France	RCT	Institutionalized persons with Alzheimer’s disease; *n* = 52 (robot-assisted *n* = 26, usual care *n* = 26)	NAO Robot (SoftBank Robotics) delivering prospective memory training using spaced retrieval technique; AUTOMATED	Robot-assisted group demonstrated significantly more positive social interaction behaviors (smiling, laughing, reaching out) during sessions than the usual care group. Both groups improved cognitively post-intervention, but no significant between-group difference was observed in cognitive outcomes, indicating no specific advantage of robot-assisted training over usual care on cognition.
5	Yun et al. (2024) [30]	South Korea	RCT	Older adults in rural South Korea (older adults with mild cognitive impairment *n* = 68, older adults with normal cognitive *n* = 55, total *n* = 123) intervention *n* = 84, control *n* = 39	AI chatbot-based mobile application; AUTOMATED	The intervention group showed significant improvements in cognitive performance compared to the control group. Higher app usage was associated with greater cognitive improvement, particularly influenced by daily mission reminders and chat-based gameplay. Both cognitively impaired and normal participants within the intervention group showed cognitive improvement, with a more pronounced effect in the impaired group.
6	Au-Yeung et al. (2024) [31]	Hong Kong	Pilot RCT	Elderly individuals with Mild Cognitive Impairment (MCI); *n* = 8 (mean age 70.1 years)	NAO robot delivering Cognitive Training (CT) and Reminiscence Therapy (RT); robot-led vs. human-led comparison measured via functional near-infrared spectroscopy (fNIRS) prefrontal cortex (dorsolateral prefrontal cortex (DLPFC)) activation; AUTOMATED (robot-led condition)	No significant difference in DLPFC activation between robot-led and human-led groups, suggesting SAR-led therapy is comparable to human-led. RT showed greater DLPFC deactivation during memory encoding but more activation during retrieval than CT. Robot-led CT may reduce performance anxiety in MCI elders. Robot Social Attributes Scale (RoSAS): 83.3% rated NAO as competent, 54.2% as warm.
7	Lee et al. (2020) [32]	Korea	Pilot RCT	Patients with Mild Cognitive Impairment (MCI); *n* = 46	Personal robot; AUTOMATED	After a 4-week home-based intervention, the robot group showed greater improvement in working memory compared to the control group. The study suggests that this robot intervention can improve working memory in MCI patients.
8	Tahan & N’kaoua (2024) [33]	France	Pilot RCT	People with Alzheimer’s disease (AD); *n* = 14	NAO Robot; AUTOMATED	Prospective Memory (PM) performance was identical when tasks were performed with the robot or with a caregiver. Engagement and attention levels were higher with the robot than with the caregiver.
9	Pino et al. (2020) [34]	Italy	Quasi-experimental	Adults with Mild Cognitive Impairment; *n* = 24 (three groups of 8)	NAO robot; AUTOMATED	Memory training with NAO resulted in increased visual gaze from patients and reinforced therapeutic behavior, reducing depressive symptoms in some cases. Unexpectedly, significant changes in prose memory and verbal fluency measures were detected. These findings suggest further research on robotics in ecological settings is necessary.
10	Amabili et al. (2022) [35]	Italy	Feasibility study	Older participants with dementia and their informal caregivers; *n* = 9 dyads	eWare system (Sensara lifestyle monitoring and Tinybot social robot); AUTOMATED (Tinybot’s AI learned and adapted, and was personalized via an app).	The eWare system showed a positive impact in supporting participants’ personal goals and improving informal caregivers’ quality of life. The impact on reducing caregivers’ burden requires further investigation. Modifications, particularly to interactivity capabilities, are needed to better meet the needs of people with dementia and ensure long-term use.
11	D’Onofrio et al. (2019) [36]	Italy	Feasibility study	People with Alzheimer’s disease; *n* = 38	MARIO companion robot (Kompa’2 robot platform) with apps (My Music, MyReminiscence, My News, My Games, My Calendar, My Family and Friends); AUTOMATED	Post-MARIO interactions showed significant improvements in resilience (Resilience Scale-14 (RS-14): *p* = 0.020). Younger participants (55–67 years) exhibited reduced depression (Cornell Scale for Depression in Dementia (CSDD): *p* = 0.033) and increased resilience (RS-14: *p* = 0.003). Older participants (68–76 years) reported greater social support (Multidimensional Scale of Perceived Social Support (MSPSS) Total: *p* = 0.016, MSPSS Fri: *p* = 0.014), and the oldest group (77–85 years) perceived increased family support (MSPSS Fam: *p* = 0.018).
12	Navarro et al. (2018) [37]	Mexico	Feasibility study	Patients with mild cognitive impairment and mild dementia; *n* = 39 (main study) +7 (Internet of Things (IoT) pilot)	Mente Activa; AUTOMATED	The system automatically generated therapy plans that were in agreement with therapists’ assessments. Experiments showed that the system could adapt therapy plans over time in response to changing levels of impairment/performance.
13	Yuan et al. (2023) [38]	USA	Feasibility study	Persons living with Alzheimer’s disease and Alzheimer’s-disease-related dementias (PLWD), mild cognitive impairment (MCI), and informal caregivers; *n* = 12	Humanoid social robot prototype; AUTOMATED	PLWD demonstrated an overall positive user experience with the RMRT, engaging in laughter and singing. Participants showed an intention to use the system. The RMRT facilitates both verbal and nonverbal social interaction, showing promise for home-based cognitive exercises.
14	Brill et al. (2025) [39]	Switzerland	Single-arm proof-of-concept/feasibility study (two-week)	Older adults with subjective cognitive decline (SCD; *n* = 12) or mild cognitive impairment (MCI; *n* = 3); *n* = 15; mean age 70.3 years	Rule-based conversational agent “Elsa” (female) / “Erik” (male) delivered via MobileCoach smartphone platform using just-in-time adaptive intervention (JITAI); AUTOMATED	Participants demonstrated 81% adherence to conversational agent-initiated turns. Technology acceptance was high across ease of use and working alliance dimensions. The study established feasibility for deploying conversational AI-based JITAI with healthy lifestyle behavioral support in older adults with SCD or MCI.
15	Meyer et al. (2026) [40]	USA	Feasibility study	Latino family caregivers of persons living with dementia; *n* = 55	NeuViCare™ web-based app with AI digital assistant “Keiko” (LLM-based) complementing the CONFIDENCE psychoeducation intervention; AUTOMATED	50.9% (28/55) of caregivers registered for the NeuViCare™ app. App users attended significantly more group sessions than non-users (mean 2.9/4 vs. 2.0/4; *p* = 0.002). Interactive AI features (Care Advisor and Resource Advisor) were infrequently used. Caregivers received an average of 21 of 30 automated text messages. App-enhanced psychoeducation showed promise for improving treatment adherence among Latino AD/Alzheimer’s disease and related dementias (ADRD) caregivers.
16	Sheehy et al. (2024) [41]	Canada	Two-stage development and feasibility study	People living with dementia (PLWD) in long-term care (LTC); Stage 1: *n* = 10 (avg age 82 years); Stage 2: *n* = 10 (avg age 87 years)	VR-based virtual companion “Kiera” presented via Meta Quest 2 head-mounted display; Stage 1: rule-based NLP; Stage 2: GPT-3.5 (OpenAI) with ElevenLabs text-to-speech (TTS); AUTOMATED	Stage 1 session lengths averaged 2:48 min with limited reciprocal conversation due to scripted responses. Stage 2 session lengths extended to up to 53:50 min with intimate and meaningful conversations across diverse topics. Six of ten Stage 2 participants expressed willingness to interact with the virtual companion again. No simulator sickness was reported. Headset weight was the primary hardware complaint.
17	Abdollahi et al. (2017) [42]	USA	Pilot study	Elderly individuals with moderate dementia and/or depression in a senior living facility (Denver, CO); *n* = 6	Ryan Companionbot (rear-projected life-like conversational robot with spoken dialogue, facial emotion recognition, cognitive games, reminiscence/photo album, music player, medication reminders); AUTOMATED	Sustained engagement over 4–6 weeks (avg. 198 dialogues/day; ~2 h 10 min/day). Exit survey: enjoyment 4.17/5, photo album 4.33/5. Caregivers confirmed mood improvement. Robot accepted as companion; interest did not decay over time. Five of six participants requested extended access.
18	Barrett et al. (2019) [43]	Ireland	Pilot study (single-group pre-post)	*n* = 10 persons with dementia in a nursing home (residential care, rural Ireland)	MARIO social robot (KOMPAI platform, 1.5 m, voice & touchscreen); personalized apps: music, reminiscence, news, games, calendar; up to 12 sessions over 4 weeks	Participants showed sustained interest and acceptance of MARIO’s appearance, sound, and applications; social engagement time increased. No statistically significant changes in quality of life (QoL), depression, or perceived social support. Demonstrates feasibility of deploying a social companion robot in a real-world nursing home; larger sample and longer intervention period recommended.
19	De Carolis et al. (2020) [44]	Italy	Pilot study	Older adults with MCI and Mild Dementia; *n* = 8	Pepper robot (SoftBank Robotics); AUTOMATED with partial human support—Pepper autonomously delivered a 3-week Cognitive Stimulation Therapy (CST) programme including motor imitation, word completion, verbal and visual-verbal associative memory, and prose memory tasks.	Participants actively engaged with Pepper, experiencing predominantly positive emotions (avg 19.33% positive vs. <3% negative per session). Seniors with lower MMSE were less happy (*r* = −0.80) but showed higher eye-gaze engagement (*r* = 0.42). Prose memory task showed very low accuracy (0.2% correct vs. 55% average for other tasks). Study terminated early due to COVID-19; no control group comparison was possible.
20	Pou-Prom et al. (2020) [45]	Canada	Pilot study	Older adults with Alzheimer’s disease (AD); *n* = 19	Ludwig; AUTOMATED	Conversations with the robot were generally well-liked and captured participant interest, despite being shorter with more misunderstandings compared to human conversations. Miscommunication did not deter participants from the experience, suggesting the robot’s potential for engagement. The robot’s ability to extract linguistic features highlights its potential as a monitoring tool for cognitive decline.
21	Ruggiano et al. (2024) [46]	United States	Pilot study	Dementia caregivers; *n* = 21	CareHeroes; AUTOMATED	Caregivers used many features of the CareHeroes app, with the chatbot being the most frequently used. Caregivers’ depression was lower at the 3-month follow-up (t_11_ = 2.03, *p* = 0.03). The pilot study demonstrated that integrating a new supportive app for caregivers as an adjunct to clinical dementia care is feasible.
22	Soler et al. (2015) [47]	Spain	Pilot study	Patients with dementia; *n* = 101 (Phase 1 nursing home), *n* = 110 (Phase 2 nursing home), *n* = 20 (Phase 1 day care), *n* = 17 (Phase 2 day care).	NAO (humanoid robot); AUTOMATED.	In the nursing home, patients in robot groups showed improvement in apathy, but the NAO group showed a decline in MMSE scores. In the day care center, there was an improvement in Neuropsychiatric Inventory (NPI) irritability and total score with the NAO robot.
23	Nakamura et al. (2020) [48]	Japan	Observational/behavioral time study (pre-post behavioral observation)	Nursing home residents (*n* = 21; 6 M, 15 W; avg age 84.7 years; avg MMSE 5.7/30) and care staff (*n* = 8)	Pepper robot (SoftBank Robotics; verbal socially assistive robot [SAR]) tele-operated to facilitate group gymnastics with talking and game applications; AUTOMATED	Resident-caregiver conversation time rate increased from 3.6% (pre-introduction) to 6.8% (Day 1) and 6.3% (Day 14) of total caregiver floor time; this increase was not statistically significant (Wilcoxon signed-rank test). Robot assumption of gymnastics demonstration role freed caregivers to provide individualized verbal care and movement assistance during group activities, representing a qualitative shift in caregiver role.
24	Iio et al. (2020) [49]	Japan	Field trial (between-participant experimental comparison)	Nursing home residents; *n* = 30 (26 F, 4 M; mean age 86.3 years, SD = 7.5); cognitive profile: 13 no dementia, 4 mild dementia, 13 advanced dementia; *n* = 24 analyzed (6 excluded: 1 hearing impairment, 5 technical failures)	Twin-robot dialogue system using CommU desktop robots (VSTONE; 304 mm high); question–answer–response (Q-A-R) dialogue model with NTT Docomo cloud ASR; one-robot (*n* = 11) vs. two-robot (*n* = 13) between-participant comparison; AUTOMATED	Average dialogue time 12 min 51 s (SD = 4 min 52 s) despite average WER = 0.778. No significant one-robot vs. two-robot difference (Mann–Whitney U = 48.0, *p* = 0.188); medium effect size (Cohen’s *d* = −0.519) favored two robots. Caregiver-rated engagement: mean 4.92/7 (SD = 1.89). Qualitative observation suggested participants with advanced dementia appeared more actively engaged in the two-robot condition. No validated cognitive or psychosocial outcome measures were employed.
25	Malhotra et al. (2025) [50]	Singapore	Development and mixed-methods pilot study (2-phase)	Informal caregivers of persons with dementia; Phase 1: *n* = 18 (caregivers *n* = 11, healthcare providers *n* = 7); Phase 2: *n* = 10 (caregivers)	CareBuddy mobile care ecosystem incorporating an LLM-powered chatbot (GPT-4/GPT-3.5, retrieval-augmented generation [RAG] architecture), GPS-based location monitoring, peer support forum, telemedicine access, and psychoeducational self-care resources; AUTOMATED	Phase 1 mean System Usability Scale (SUS) scores improved from 65.4 (Round 1) to 73.8 (Round 3), exceeding the benchmark of 68. Phase 2 Mobile Application Usability Questionnaire (MAUQ) overall mean score was 95.4 (SD 8.5), with high ratings in ease of use (mean 24.1), interface and satisfaction (mean 40.3), and usefulness (mean 31.0). Caregivers valued the AI chatbot, peer support forum, and dementia management content. An ongoing trial is evaluating the app’s effectiveness in improving caregiver outcomes.
26	Morris et al. (2024) [51]	USA	Usability study	Persons with Alzheimer’s disease or related dementia (PwADRD); *n* = 15	Music intervention Using Socially Engaging robotics (MUSE) system with Pepper robot; AUTOMATED	PwADRD were very accepting of the MUSE system, including the social robot, musical activities, and applications. Areas for improvement identified included system volume and visibility.
27	Akinrintoyo and Salomons (2025) [52]	United Kingdom	Design & usability study	Dementia professionals (*n* = 3) and Persons with dementia (*n* = 5)	Socially assistive robotic system; AUTOMATED	The PwDs enjoyed using the system and were willing to adopt its use over the long term. A shortcoming was the system’s speech-to-text capabilities, which frequently failed to understand the PwDs. Users found the system easy to use and beneficial for cognitive stimulation.
28	Espinoza et al. (2025) [53]	Peru/United Kingdom	Multi-phase design and usability study (stakeholder engagement, design, usability evaluation, field study)	Dementia caregivers in Peru; *n* = 7	“Ana” chatbot: Version 1 (Ana-V1): rule-based NLP; Version 2 (Ana-V2): generative AI powered by GPT-3.5; delivered via WhatsApp; AUTOMATED	Ana-V2 was significantly preferred over Ana-V1 in understanding, empathy, and knowledgeability (Wilcoxon signed-rank test, *p* < 0.05). Caregivers preferred the generative AI chatbot despite concerns about inaccurate information. WhatsApp was identified as the optimal accessibility channel for resource-limited settings in Peru.
29	Mahmoudi Asl et al. (2024) [54]	Spain	Mixed-method usability study (controlled laboratory setting)	Individuals with mild dementia (MD; *n* = 5) and mild cognitive impairment (MCI; *n* = 5) from INTRAS Foundation Memory Clinic, Zamora; *n* = 10; mean age 77.3 years	MINI social robot: desktop robotic companion with speech recognition, tablet display, touch sensors, and fur-covered body; AUTOMATED	Mean System Usability Scale (SUS) score was 68.3, categorized as “good” usability. Primary usability challenges included speech recognition failures and difficulty in switching between verbal and tactile interaction modalities, particularly pronounced in the mild dementia group.
30	Nasiri and Fulda (2024) [55]	USA	Design & usability study	Caregivers of people with dementia; *n* = 24	Language-Model-Based Chatbot; AUTOMATED	The chatbot was designed to consider user personality profiles and emotions, aiming to enhance engagement. The study focused on the design and usability aspects of creating such a conversational agent for potential future applications in cognitive support.
31	Cheng & Ng (2025) [56]	China (Hong Kong)	Acceptability Study	Family caregivers of people with dementia; *n* = 21	PDC30 Chatbot; AUTOMATED	Caregivers found the PDC30 Chatbot highly user-friendly, helpful, and easy to understand, reporting high satisfaction and a strong recommendation rate. The chatbot provided context-sensitive advice, performing slightly better than alternatives when questions conveyed significant psychological distress. The majority of users engaged with the chatbot more than once daily during the two-week trial.
32	Bai et al. (2025) [57]	Taiwan	Cross-sectional comparative study	Formal dementia caregivers; *n* = 120	Social robot and tablet; AUTOMATED	Caregivers generally favored the social robot over the tablet, reporting higher overall User Experience Questionnaire (UEQ) scores, particularly in enjoyment, friendliness, clarity, organization, interest, and innovation. Technology Acceptance Model (TAM) results indicated higher total scores for the robot in perceived usefulness, ease of use, attitudes, and behavioral intentions.
33	Stara et al. (2021) [58]	Italy	Exploratory Study	Older adults living with dementia; *n* = 20	Anne; AUTOMATED	The usability of the system achieved an encouraging score, and half of the sample recognized a role for the agent Anne. Technical problems related to speech recognition negatively impacted user intention to use, adaptiveness, usefulness, and trust. The touch screen modality was positively used by patients with dementia.
34	Curumsing et al. (2025) [59]	Australia	Case study	Dementia caregivers (carers of people living with dementia/cognitive impairment); *n* = 23	Ted (embodied conversational agent); AUTOMATED	Caregivers developed an emotional connection with Ted and retained learning after 8 weeks. They implemented learned communication principles into their practice, which were well received by people living with dementia.
35	Nagata et al. (2026) [60]	Japan	Case report	Older women with mild cognitive impairment or late-onset psychosis; *n* = 5	RoBoHoN, Sharp; AUTOMATED	Usability assessments showed high satisfaction and ease of use among participants. Standardized psychological scales did not show consistent improvements, but four out of five participants expressed a desire to continue using the robot. The study suggests feasibility and potential long-term acceptability of companion robots for cognitively challenged older adults in home settings.
36	Whelan et al. (2021) [61]	Ireland	Multiple case study	People with dementia *n* = 10, caregivers *n* = 6, and relatives *n* = 7	MARIO; AUTOMATED with partial human assistance	The resilience of eight out of ten people with dementia was supported during interactions with MARIO. MARIO sessions increased wellbeing by providing meaningful activity that reinforced positive self-concept. Social robots need greater capability to interpret and respond to emotional needs to benefit resilience without a facilitator.
37	Carós et al. (2020) [62]	Spain	Usability Study	People with MCI or early-stage Alzheimer’s disease and age >60 *n* = 2, Healthy adult who age >60 *n* = 2	Elisabot; AUTOMATED	This paper presents a dialogue system that uses user photos to generate questions about their life, aiming to automate reminiscence therapy. The system is designed to be intuitive and accessible via smartphones and laptops for individuals with mild cognitive impairment or early-stage Alzheimer’s disease. It utilizes deep learning for question generation from images and a chatbot model for conversational responses. All users are enjoyed with doing therapy with Elisabot. While users with mild cognitive impairment found it challenging as they have to make effort to remember the answers for some of the generated questions.
38	Cruz-Sandoval et al. (2020) [63]	Mexico	Naturalistic Crossover Study	Persons with dementia; *n* = 8	Eva; AUTOMATED	The study reported a statistically significant decrease in the total Neuropsychiatric Inventory—Nursing Home version (NPI-NH) score for participants after the intervention. Specific symptoms like delusions, agitation/aggression, and euphoria/exaltation also showed statistically significant decreases. Caregiver interviews indicated positive short-term effects and some lasting behavioral changes.
39	Hasan et al. (2024) [64]	USA	Usability Study	ADRD caregivers; *n* = 20	ADQueryAid; AUTOMATED	ADQueryAid demonstrated superior usability and provided more personalized and contextually relevant information compared to a baseline model (ChatGPT 3.5). The system has the potential to address the unique challenges faced by caregivers, particularly those with limited medical knowledge.
40	Kang et al. (2025) [65]	Republic of Korea	Quasi-experimental study	Individuals with dementia; *n* = 80	CLOVA CareCall; AUTOMATED	After the AI care call intervention, Geriatric Depression Scale (GDS) scores decreased significantly, and memory scores increased significantly. There were no significant changes in attention or language scores. Interaction effects between age and sex, and age and education were observed for attention and memory score changes.

Note. AD = Alzheimer’s disease; ADRD = Alzheimer’s disease and related dementias; AI = artificial intelligence; ASR = automatic speech recognition; BPSD = behavioural and psychological symptoms of dementia; CSDD = Cornell Scale for Depression in Dementia; CST = cognitive stimulation therapy; CT = cognitive training; DLPFC = dorsolateral prefrontal cortex; ECA = embodied conversational agent; fNIRS = functional near-infrared spectroscopy; GDS = Geriatric Depression Scale; IoT = Internet of Things; JITAI = just-in-time adaptive intervention; LLM = large language model; LTC = long-term care; MAUQ = Mobile Application Usability Questionnaire; MCI = mild cognitive impairment; MD = mild dementia; MMSE = Mini-Mental State Examination; MSPSS = Multidimensional Scale of Perceived Social Support; MUSE = Music intervention Using Socially Engaging robotics; NLP = natural language processing; NPI = Neuropsychiatric Inventory; NPI-NH = Neuropsychiatric Inventory–Nursing Home version; NPI-Q = Neuropsychiatric Inventory Questionnaire; PLWD = people living with dementia; PM = prospective memory; PwADRD = persons with Alzheimer’s disease or related dementia; PwD = people with dementia; Q-A-R = question–answer–response; QoL = quality of life; RAG = retrieval-augmented generation; RCT = randomized controlled trial; RMRT = robot-mediated reminiscence therapy; RoB = risk of bias; RoSAS = Robot Social Attributes Scale; RS-14 = Resilience Scale-14; RT = reminiscence therapy; SAR = socially assistive robot; SCD = subjective cognitive decline; SD = standard deviation; SUS = System Usability Scale; TAM = Technology Acceptance Model; TTS = text-to-speech; UEQ = User Experience Questionnaire; VR = virtual reality; WER = word error rate.

**Table 5:** Synthesis of evidence by conversational AI modality.

Intervention Type	Effects	Strengths	Limitations	Representative Studies
**Text-based Chatbot** *n* = 12 (30.0%) **Systems:** *PDC30, CareBuddy, CareHeroes, Ana, Elisabot, ADQueryAid*	–Cognitive improvement in MCI with daily chatbot use [30] –Reduced caregiver depression and burden at 1-month [26,46] –LLM versions significantly preferred over rule-based on empathy and understanding [53] –High usability (SUS > 68; MAUQ 95.4) and adherence rates across caregiver platforms	–Low cost; deployable via smartphone or web without specialist hardware –Familiar interfaces (WhatsApp, apps) lower adoption barriers –LLM enables personalized, open-ended dialogue –Scalable for caregiver psychoeducation and support	–Few RCTs; predominantly feasibility studies with short follow-up (2–4 weeks) –LLM accuracy and safety unverified for clinical advice –Limited direct evidence in persons with dementia (caregiver-focused) –Privacy risks with cloud-based processing	[26,30,39,50,53]
**Voice-based Chatbot** *n* = 1 (2.5%) *Systems: CLOVA CareCall*	–Significant GDS depression score reduction post-intervention [65] –Significant memory score improvement; no change in attention or language –Interaction effects of age, sex, and education on cognitive outcomes observed	–Telephone-only delivery; no device or digital literacy required –Eliminates hardware and internet access barriers –Scalable at low cost via existing infrastructure	–Single study (*n* = 80); insufficient for generalizable conclusions –No visual or tactile feedback; limited interaction richness –Speech recognition challenges for dysarthric or accented speech –Critical evidence gap requiring dedicated research	[65]
**Multimodal Chatbot** *n* = 3 (7.5%) **Systems:** *Kiera (VR), Anne (touchscreen), Ted (embodied agent)*	–LLM upgrade extended conversation duration 18-fold (2:48→53:50 min) [41] –Retained behavioral learning and emotional connection at 8 weeks [59] –Touchscreen positively used by PwD despite speech recognition failures [58]	–Visual, verbal, and touch channels support varied interaction preferences –VR enables immersive reminiscence environments –LLM integration substantially enhances conversational naturalness	–Very small evidence base (*n* = 3); no RCTs –Hardware cost, headset weight, and VR setup complexity limit uptake –Speech recognition failures disproportionately affect PwD –No standardized clinical outcome measurement	[41,58,59]
**Socially Assistive Robot (SAR)** *n* = 24 (60.0%) **Systems:** *NAO, Pepper, MARIO, Kabochan, PIO, CommU, RoBoHoN*	–Significant cognitive improvement in RCTs [28,32] –Robot-led therapy comparable to human-led on fNIRS-measured DLPFC activation [31] –Significant neuropsychiatric symptom and caregiver distress reduction [27] –Increased social engagement, resilience, and positive affect across care settings [36,61] –Engagement sustained over 4–6 weeks with no interest decay [42]	–Physical embodiment enhances social presence and emotional engagement –Multi-modal interaction (speech, gesture, gaze, touch) supports severe cognitive decline –Largest evidence base (*n* = 24, incl. 6 RCTs); feasible across home, day care, and nursing home –Can serve as companion, cognitive trainer, and caregiver assistant simultaneously	–High device cost, maintenance burden, and technical failure risk –Speech recognition commonly fails (WER = 0.778; Iio et al., 2020) [49] –Mixed cognitive RCT outcomes; between-group differences not always significant –Study heterogeneity and small single-site samples preclude meta-analysis	[27,28,31,33,36,42]

Note. AI = artificial intelligence; DLPFC = dorsolateral prefrontal cortex; fNIRS = functional near-infrared spectroscopy; GDS = Geriatric Depression Scale; LLM = large language model; MAUQ = Mobile Application Usability Questionnaire; MCI = mild cognitive impairment; PwD = people with dementia; RCT = randomized controlled trial; SAR = socially assistive robot; SUS = System Usability Scale; VR = virtual reality; WER = word error rate; → indicates change from baseline to follow-up.

**Table 6:** Conversational AI and large language model risks mapped to research and governance priorities, with supporting studies from the included evidence base.

Risk	Description	Supporting Studies
**Hallucination/factual inaccuracy**	Generative models can produce plausible but false information; caregivers may act on inaccurate AI-generated clinical guidance.	[26,55,64]
**Privacy/data security**	Cloud processing and storage of sensitive conversational and clinical data from vulnerable users.	[46,50,53]
**Informed consent/capacity**	Variable and fluctuating capacity of people with dementia to consent to AI interaction.	[27,47]
**User safety/over-reliance & attachment**	Vulnerable users may over-trust, become dependent on, or form deceptive attachments to AI companionship.	[27,60,61]
**Misinformation propagation**	Incorrect health information can be amplified at scale across users and caregivers.	[55,64]
**Speech recognition/interaction failure**	ASR errors, voice-control failure, and language/dialect mismatch degrade usefulness, trust, and engagement.	[27,49,52,58]
**Unproven clinical efficacy of LLM systems**	Engagement and conversation-depth gains from LLMs are not matched by controlled evidence of clinical benefit.	[26,28,30]

Note. ASR = automatic speech recognition; BPSD = behavioral and psychological symptoms of dementia; DLPFC = dorsolateral prefrontal cortex; fNIRS = functional near-infrared spectroscopy; LLM = large language model; SAR = socially assistive robot; SUS = System Usability Scale; UCLA-LS = UCLA Loneliness Scale; WER = word error rate.

## Data Availability

No new data were created or analyzed in this study. Data sharing is not applicable to this article.

## Supplementary Materials

The following supporting information can be downloaded at: https://www.mdpi.com/article/10.3390/healthcare14142106/s1, Supplementary File S1: PRISMA 2020 Checklist; Supplementary File S2: Full Database Search Strategies.

## Abbreviations

The following abbreviations are used in this manuscript:

AD	Alzheimer’s disease
ADAS-cog	Alzheimer’s Disease Assessment Scale–Cognitive Subscale
AI	artificial intelligence
ASR	automatic speech recognition
BPSD	behavioral and psychological symptoms of dementia
CIRCA	Computer Interactive Reminiscence and Conversation Aid
CST	cognitive stimulation therapy
DLPFC	dorsolateral prefrontal cortex
fNIRS	functional near-infrared spectroscopy
GDS	Geriatric Depression Scale
GRADE	Grading of Recommendations, Assessment, Development, and Evaluations
K-MMSE 2	Korean Mini-Mental State Examination, 2nd Edition
LLM	large language model
MAUQ	Mobile Application Usability Questionnaire
MCI	mild cognitive impairment
MMSE	Mini-Mental State Examination
MoCA	Montreal Cognitive Assessment
NLP	natural language processing
NPI	Neuropsychiatric Inventory
NPI-NH	Neuropsychiatric Inventory—Nursing Home version
NPI-Q	Neuropsychiatric Inventory Questionnaire
PHQ-9	Patient Health Questionnaire-9
PICOS	Population, Intervention, Comparator, Outcomes, Study Design
PRISMA	Preferred Reporting Items for Systematic Reviews and Meta-Analyses
PROSPERO	International Prospective Register of Systematic Reviews
PwD	persons with dementia
RCT	randomized controlled trial
RoB 2	Cochrane Risk of Bias Tool version 2
ROBINS-I	Risk of Bias in Non-Randomized Studies of Interventions
RS-14	Resilience Scale-14
SAR	socially assistive robot
SUS	System Usability Scale
TAM	Technology Acceptance Model
UCLA-LS	UCLA Loneliness Scale
UEQ	User Experience Questionnaire
VR	virtual reality
WER	word error rate
ZBI	Zarit Burden Interview
