# Supplementary material for: Conversational AI in Cognitive and Social Training for People with Dementia: A Systematic Review
Source: Healthcare (Basel). 2026 Jul 14;14(14):2106. doi: 10.3390/healthcare14142106 (PMC13410334; doi:10.3390/healthcare14142106)
Supplement: Supplementary file 1 [file healthcare-14-02106-s001.zip › Supplementary S1- PRISMA 2020 Checklist.pdf]

PRISMA 2020 Checklist

Section and Topic	Item #	Checklist item	Location where item is reported
**TITLE**			
Title	1	Identify the report as a systematic review.	Title page — "A Systematic Review" stated in full title
**ABSTRACT**			
Abstract	2	See the PRISMA 2020 for Abstracts checklist.	Abstract section (Page 1) — structured under Background, Methods, Results, Conclusions
**INTRODUCTION**			
Rationale	3	Describe the rationale for the review in the context of existing knowledge.	Section 1, Introduction, paragraphs 1–4 (Pages 1–2)
Objectives	4	Provide an explicit statement of the objective(s) or question(s) the review addresses.	Section 1, Introduction, final paragraph (Page 2) — "This review aims to synthesise the current research..."
**METHODS**			
Eligibility criteria	5	Specify the inclusion and exclusion criteria for the review and how studies were grouped for the syntheses.	Section 2.1.2; Table 2 (PICOS Eligibility Criteria); Table 3 (Modality Definitions) (Pages 3–5)
Information sources	6	Specify all databases, registers, websites, organisations, reference lists and other sources searched or consulted to identify studies. Specify the date when each source was last searched or consulted.	Section 2.1.1 (Page 3) — six databases listed (PubMed, Embase, Web of Science, Scopus, IEEE Xplore, ACM Digital Library); final search 6 May 2026
Search strategy	7	Present the full search strategies for all databases, registers and websites, including any filters and limits used.	Table 1 (Page 3) — full Boolean search strings; Supplementary File S2 — complete per-database strategies
Selection process	8	Specify the methods used to decide whether a study met the inclusion criteria of the review, including how many reviewers screened each record and each report retrieved, whether they worked independently, and if applicable, details of automation tools used in the process.	Section 2.1.3 (Page 5) — two independent reviewers (K.K.C. & P.N.); title/abstract then full-text screening; Cohen's kappa reported; discrepancies resolved by discussion or third reviewer
Data collection process	9	Specify the methods used to collect data from reports, including how many reviewers collected data from each report, whether they worked independently, any processes for obtaining or confirming data from study investigators, and if applicable, details of automation tools used in the process.	Section 2.1.3 (Page 5) — structured data extraction form; two independent reviewers; fields specified
Data items	10a	List and define all outcomes for which data were sought. Specify whether all results that were compatible with each outcome	Section 2.4 (Page 6) — four

PRISMA 2020 Checklist

Section and Topic	Item #	Checklist item	Location where item is reported
		domain in each study were sought (e.g. for all measures, time points, analyses), and if not, the methods used to decide which results to collect.	pre-specified outcome domains (cognitive function; social engagement; caregiver burden; system acceptability); all reported timepoints eligible
	10b	List and define all other variables for which data were sought (e.g. participant and intervention characteristics, funding sources). Describe any assumptions made about any missing or unclear information.	Section 2.1.3 (Page 5) — study characteristics, participant details, chatbot features, application context, primary outcomes, and challenges listed
Study risk of bias assessment	11	Specify the methods used to assess risk of bias in the included studies, including details of the tool(s) used, how many reviewers assessed each study and whether they worked independently, and if applicable, details of automation tools used in the process.	Section 2.3 (Pages 5–6) — RoB 2 for RCTs (5 domains); ROBINS-I for non-randomised studies (7 domains); duplicate assessment; disagreements resolved by discussion
Effect measures	12	Specify for each outcome the effect measure(s) (e.g. risk ratio, mean difference) used in the synthesis or presentation of results.	Not reported — narrative synthesis only; no meta-analysis performed (see Section 2.2, Page 5)
Synthesis methods	13a	Describe the processes used to decide which studies were eligible for each synthesis (e.g. tabulating the study intervention characteristics and comparing against the planned groups for each synthesis (item #5)).	Section 2.2 (Page 5) — all included studies eligible for narrative synthesis; grouped by pre-specified modality taxonomy (Table 3)
	13b	Describe any methods required to prepare the data for presentation or synthesis, such as handling of missing summary statistics, or data conversions.	Section 2.2 (Page 5) — narrative synthesis; no formal data conversions; heterogeneity precluded meta-analysis
	13c	Describe any methods used to tabulate or visually display results of individual studies and syntheses.	Section 2.2 (Page 5); Table 4 (Page 11) — synthesis by modality; Figures 3–4 — risk of bias summary plots
	13d	Describe any methods used to synthesize results and provide a rationale for the choice(s). If meta-analysis was performed, describe the model(s), method(s) to identify the presence and extent of statistical heterogeneity, and software package(s) used.	Section 2.2 (Page 5) — narrative synthesis; rationale stated (heterogeneity of interventions, designs, and outcome measures precluded meta-analysis)

PRISMA 2020 Checklist

Section and Topic	Item #	Checklist item	Location where item is reported
	13e	Describe any methods used to explore possible causes of heterogeneity among study results (e.g. subgroup analysis, meta-regression).	Not reported — no formal heterogeneity analysis conducted; noted as limitation in Section 4.3 (Page 17)
	13f	Describe any sensitivity analyses conducted to assess robustness of the synthesized results.	Not reported — no sensitivity analyses conducted
Reporting bias assessment	14	Describe any methods used to assess risk of bias due to missing results in a synthesis (arising from reporting biases).	Section 4.3, Limitations (Page 17) — reporting bias not formally assessed; acknowledged as limitation given likely positive-result publication bias
Certainty assessment	15	Describe any methods used to assess certainty (or confidence) in the body of evidence for an outcome.	Section 4.3, Limitations (Page 17) — GRADE not applied; acknowledged as limitation per registered protocol
**RESULTS**			
Study selection	16a	Describe the results of the search and selection process, from the number of records identified in the search to the number of studies included in the review, ideally using a flow diagram.	Section 3.1 (Page 7); Figure 1 — PRISMA 2020 flow diagram (3213 records → 40 included studies)
	16b	Cite studies that might appear to meet the inclusion criteria, but which were excluded, and explain why they were excluded.	Section 3.1 (Page 7) — 87 full-text exclusions with reasons by category (ineligible design n=31; ineligible intervention n=13; ineligible population n=18; insufficient data n=16; irrelevant outcome n=9)
Study characteristics	17	Cite each included study and present its characteristics.	Section 3.2 (Pages 7–8); Supplementary File S3 — full study characteristics extraction table (40 studies)
Risk of bias in studies	18	Present assessments of risk of bias for each included study.	Section 3.4 (Pages 9–10); Figure 3 — RoB 2 summary; Figure 4 — ROBINS-I summary
Results of individual studies	19	For all outcomes, present, for each study: (a) summary statistics for each group (where appropriate) and (b) an effect estimate and its precision (e.g. confidence/credible interval), ideally using structured tables or plots.	Sections 3.5–3.8 (Pages 10–15) — study-level results reported narratively for each outcome domain

PRISMA 2020 Checklist

Section and Topic	Item #	Checklist item	Location where item is reported
Results of syntheses	20a	For each synthesis, briefly summarise the characteristics and risk of bias among contributing studies.	Table 4 (Page 11); Sections 3.5–3.8 (Pages 10–15) — modality-level synthesis with RoB context
	20b	Present results of all statistical syntheses conducted. If meta-analysis was done, present for each the summary estimate and its precision (e.g. confidence/credible interval) and measures of statistical heterogeneity. If comparing groups, describe the direction of the effect.	Not applicable — no meta-analysis performed; narrative synthesis only
	20c	Present results of all investigations of possible causes of heterogeneity among study results.	Not applicable — no formal heterogeneity investigation conducted
	20d	Present results of all sensitivity analyses conducted to assess the robustness of the synthesized results.	Not applicable — no sensitivity analyses conducted
Reporting biases	21	Present assessments of risk of bias due to missing results (arising from reporting biases) for each synthesis assessed.	Section 4.3, Limitations (Page 17) — reporting bias not formally assessed; acknowledged as limitation
Certainty of evidence	22	Present assessments of certainty (or confidence) in the body of evidence for each outcome assessed.	Section 4.3, Limitations (Page 17) — GRADE not applied; acknowledged as limitation per registered protocol
**DISCUSSION**			
Discussion	23a	Provide a general interpretation of the results in the context of other evidence.	Section 4.1, Principal Findings (Pages 15–16) — four key findings interpreted against existing literature
	23b	Discuss any limitations of the evidence included in the review.	Section 4.3, Limitations (Pages 16–17) — heterogeneity, small RCT evidence base, RoB distribution, single voice-based study
	23c	Discuss any limitations of the review processes used.	Section 4.3, Limitations (Pages 16–17) — English-language restriction, no GRADE, no reporting bias assessment, narrative synthesis
	23d	Discuss implications of the results for practice, policy, and future research.	Section 4.2, Clinical Implications (Pages 16–17); Section 5, Conclusions (Page 17) — clinical

PRISMA 2020 Checklist

Section and Topic	Item #	Checklist item	Location where item is reported
			deployment priorities and future research directions
**OTHER INFORMATION**			
Registration and protocol	24a	Provide registration information for the review, including register name and registration number, or state that the review was not registered.	Abstract, Methods section (Page 1); Section 2 (Page 3) — PROSPERO CRD420261333625
	24b	Indicate where the review protocol can be accessed, or state that a protocol was not prepared.	Section 2 (Page 3) — PROSPERO CRD420261333625; accessible at www.crd.york.ac.uk/prospero
	24c	Describe and explain any amendments to information provided at registration or in the protocol.	Section 2.2 — one amendment logged: the search strategy was revised (removal of “online programs”) to improve precision, with a corresponding extension of the review timeline; PROSPERO record updated accordingly
Support	25	Describe sources of financial or non-financial support for the review, and the role of the funders or sponsors in the review.	Back matter, Funding section (Page 18) — "This research received no external funding"
Competing interests	26	Declare any competing interests of review authors.	Back matter, Conflicts of Interest section (Page 18) — "The authors declare no conflicts of interest"
Availability of data, code and other materials	27	Report which of the following are publicly available and where they can be found: template data collection forms; data extracted from included studies; data used for all analyses; analytic code; any other materials used in the review.	Back matter, Supplementary Materials and Data Availability Statement (Page 18) — Supplementary Files S1–S3 listed; no new data created

From: Page MJ, McKenzie JE, Bossuyt PM, Boutron I, Hoffmann TC, Mulrow CD, et al. The PRISMA 2020 statement: an updated guideline for reporting systematic reviews. BMJ 2021;372:n71. doi: 10.1136/bmj.n71. This work is licensed under CC BY 4.0. To view a copy of this license, visit https://creativecommons.org/licenses/by/4.0/
