# Supplementary material for: Conversational AI in Cognitive and Social Training for People with Dementia: A Systematic Review
Source: Healthcare (Basel). 2026 Jul 14;14(14):2106. doi: 10.3390/healthcare14142106 (PMC13410334; doi:10.3390/healthcare14142106)
Supplement: Supplementary file 1 [file healthcare-14-02106-s001.zip › Supplementary S2 - Full Search Strategy.pdf]

Search Strategy

Conversational AI in Cognitive and Social Training for People with Dementia: A Systematic Review

Search dates: All databases were searched on 26 June 2026

Source	Search Strategy	Hits Retrieved
**1. PubMed**	1. chatbot[Title/Abstract]	**295**
	2. "conversational agent"[Title/Abstract]	
	3. "dialogue system"[Title/Abstract]	
	4. "virtual assistant"[Title/Abstract]	
	5. "conversational AI"[Title/Abstract]	
	6. "social robot*"[Title/Abstract]	
	7. "embodied conversational agent*"[Title/Abstract]	
	8. "large language model*"[Title/Abstract]	
	9. LLM*[Title/Abstract]	
	10. ChatGPT[Title/Abstract]	
	11. "digital companion*"[Title/Abstract]	
	12. "voice assistant*"[Title/Abstract]	
	13. dementia[Title/Abstract]	
	14. "Alzheimer's disease"[Title/Abstract]	
	15. "cognitive impairment"[Title/Abstract]	
	16. MCI[Title/Abstract]	
	17. "older adult*"[Title/Abstract]	
	18. elder*[Title/Abstract]	
	19. aging[Title/Abstract]	
	20. ageing[Title/Abstract]	
	21. elderly[Title/Abstract]	
	22. "cognitive decline"[Title/Abstract]	
	23. "memory loss"[Title/Abstract]	
	24. "cognitive training"[Title/Abstract]	
	25. "social engagement"[Title/Abstract]	
	26. intervention[Title/Abstract]	
	27. therapy[Title/Abstract]	
	28. reminiscence*[Title/Abstract]	
	29. "cognitive stimulation"[Title/Abstract]	
	30. caregiver*[Title/Abstract]	
	31. 1 OR 2 OR 3 OR 4 OR 5 OR 6 OR 7 OR 8 OR 9 OR 10 OR 11 OR 12	
	32. 13 OR 14 OR 15 OR 16 OR 17 OR 18 OR 19 OR 20 OR 21 OR 22 OR 23	
	33. 24 OR 25 OR 26 OR 27 OR 28 OR 29 OR 30	
	34. 31 AND 32 AND 33	
**2. Embase**	1. chatbot:ab,ti	**311**
	2. 'conversational agent':ab,ti	
	3. 'dialogue system':ab,ti	
	4. 'virtual assistant':ab,ti	
	5. 'conversational AI':ab,ti	
	6. 'social robot*':ab,ti	
	7. 'embodied conversational agent*':ab,ti	
	8. 'large language model*':ab,ti	
	9. LLM*:ab,ti	

	10. ChatGPT:ab,ti	
	11. 'digital companion*':ab,ti	
	12. 'voice assistant*':ab,ti	
	13. dementia:ab,ti	
	14. 'Alzheimer's disease':ab,ti	
	15. 'cognitive impairment':ab,ti	
	16. MCI:ab,ti	
	17. 'older adult*':ab,ti	
	18. elder*:ab,ti	
	19. aging:ab,ti	
	20. ageing:ab,ti	
	21. elderly:ab,ti	
	22. 'cognitive decline':ab,ti	
	23. 'memory loss':ab,ti	
	24. 'cognitive training':ab,ti	
	25. 'social engagement':ab,ti	
	26. intervention:ab,ti	
	27. therapy:ab,ti	
	28. reminiscence*:ab,ti	
	29. 'cognitive stimulation':ab,ti	
	30. caregiver*:ab,ti	
	31. 1 OR 2 OR 3 OR 4 OR 5 OR 6 OR 7 OR 8 OR 9 OR 10 OR 11 OR 12	
	32. 13 OR 14 OR 15 OR 16 OR 17 OR 18 OR 19 OR 20 OR 21 OR 22 OR 23	
	33. 24 OR 25 OR 26 OR 27 OR 28 OR 29 OR 30	
	34. 31 AND 32 AND 33	
**3. Web of Science**	1. TI=(chatbot) OR AB=(chatbot) OR AK=(chatbot)	**1196**
	2. TI="conversational agent" OR AB="conversational agent" OR AK="conversational agent"	
	3. TI="dialogue system" OR AB="dialogue system" OR AK="dialogue system"	
	4. TI="virtual assistant" OR AB="virtual assistant" OR AK="virtual assistant"	
	5. TI="conversational AI" OR AB="conversational AI" OR AK="conversational AI"	
	6. TI="social robot*" OR AB="social robot*" OR AK="social robot*"	
	7. TI="embodied conversational agent*" OR AB="embodied conversational agent*" OR AK="embodied conversational agent*"	
	8. TI="large language model*" OR AB="large language model*" OR AK="large language model*"	
	9. TI=(LLM*) OR AB=(LLM*) OR AK=(LLM*)	
	10. TI=(ChatGPT) OR AB=(ChatGPT) OR AK=(ChatGPT)	
	11. TI="digital companion*" OR AB="digital companion*" OR AK="digital companion*"	
	12. TI="voice assistant*" OR AB="voice assistant*" OR AK="voice assistant*"	
	13. TI=(dementia) OR AB=(dementia) OR AK=(dementia)	
	14. TI="Alzheimer's disease" OR AB="Alzheimer's disease" OR AK="Alzheimer's disease"	
	15. TI="cognitive impairment" OR AB="cognitive impairment" OR AK="cognitive impairment"	
	16. TI=(MCI) OR AB=(MCI) OR AK=(MCI)	
	17. TI="older adult*" OR AB="older adult*" OR AK="older adult*"	
	18. TI=(elder*) OR AB=(elder*) OR AK=(elder*)	

	19. TI=(aging) OR AB=(aging) OR AK=(aging)	
	20. TI=(ageing) OR AB=(ageing) OR AK=(ageing)	
	21. TI=(elderly) OR AB=(elderly) OR AK=(elderly)	
	22. TI="cognitive decline" OR AB="cognitive decline" OR AK="cognitive decline"	
	23. TI="memory loss" OR AB="memory loss" OR AK="memory loss"	
	24. TI="cognitive training" OR AB="cognitive training" OR AK="cognitive training"	
	25. TI="social engagement" OR AB="social engagement" OR AK="social engagement"	
	26. TI=(intervention) OR AB=(intervention) OR AK=(intervention)	
	27. TI=(therapy) OR AB=(therapy) OR AK=(therapy)	
	28. TI=(reminiscence*) OR AB=(reminiscence*) OR AK=(reminiscence*)	
	29. TI="cognitive stimulation" OR AB="cognitive stimulation" OR AK="cognitive stimulation"	
	30. TI=(caregiver*) OR AB=(caregiver*) OR AK=(caregiver*)	
	31. 1 OR 2 OR 3 OR 4 OR 5 OR 6 OR 7 OR 8 OR 9 OR 10 OR 11 OR 12	
	32. 13 OR 14 OR 15 OR 16 OR 17 OR 18 OR 19 OR 20 OR 21 OR 22 OR 23	
	33. 24 OR 25 OR 26 OR 27 OR 28 OR 29 OR 30	
	34. 31 AND 32 AND 33	
**4. Scopus**	1. TITLE-ABS-KEY("chatbot")	**1270**
	2. TITLE-ABS-KEY("conversational agent")	
	3. TITLE-ABS-KEY("dialogue system")	
	4. TITLE-ABS-KEY("virtual assistant")	
	5. TITLE-ABS-KEY("conversational AI")	
	6. TITLE-ABS-KEY("social robot*")	
	7. TITLE-ABS-KEY("embodied conversational agent*")	
	8. TITLE-ABS-KEY("large language model*")	
	9. TITLE-ABS-KEY("LLM*")	
	10. TITLE-ABS-KEY("ChatGPT")	
	11. TITLE-ABS-KEY("digital companion*")	
	12. TITLE-ABS-KEY("voice assistant*")	
	13. TITLE-ABS-KEY("dementia")	
	14. TITLE-ABS-KEY("Alzheimer's disease")	
	15. TITLE-ABS-KEY("cognitive impairment")	
	16. TITLE-ABS-KEY("MCI")	
	17. TITLE-ABS-KEY("older adult*")	
	18. TITLE-ABS-KEY("elder*")	
	19. TITLE-ABS-KEY("aging")	
	20. TITLE-ABS-KEY("ageing")	
	21. TITLE-ABS-KEY("elderly")	
	22. TITLE-ABS-KEY("cognitive decline")	
	23. TITLE-ABS-KEY("memory loss")	
	24. TITLE-ABS-KEY("cognitive training")	
	25. TITLE-ABS-KEY("social engagement")	
	26. TITLE-ABS-KEY("intervention")	
	27. TITLE-ABS-KEY("therapy")	
	28. TITLE-ABS-KEY("reminiscence*")	
	29. TITLE-ABS-KEY("cognitive stimulation")	
	30. TITLE-ABS-KEY("caregiver*")	

	31. 1 OR 2 OR 3 OR 4 OR 5 OR 6 OR 7 OR 8 OR 9 OR 10 OR 11 OR 12	
	32. 13 OR 14 OR 15 OR 16 OR 17 OR 18 OR 19 OR 20 OR 21 OR 22 OR 23	
	33. 24 OR 25 OR 26 OR 27 OR 28 OR 29 OR 30	
	34. 31 AND 32 AND 33	
**5. IEEE Xplore**	1. TITLE-ABS-KEY("chatbot")	**117**
	2. TITLE-ABS-KEY("conversational agent")	
	3. TITLE-ABS-KEY("dialogue system")	
	4. TITLE-ABS-KEY("virtual assistant")	
	5. TITLE-ABS-KEY("conversational AI")	
	6. TITLE-ABS-KEY("social robot*")	
	7. TITLE-ABS-KEY("embodied conversational agent*")	
	8. TITLE-ABS-KEY("large language model*")	
	9. TITLE-ABS-KEY("LLM*")	
	10. TITLE-ABS-KEY("ChatGPT")	
	11. TITLE-ABS-KEY("digital companion*")	
	12. TITLE-ABS-KEY("voice assistant*")	
	13. TITLE-ABS-KEY("dementia")	
	14. TITLE-ABS-KEY("Alzheimer's disease")	
	15. TITLE-ABS-KEY("cognitive impairment")	
	16. TITLE-ABS-KEY("MCI")	
	17. TITLE-ABS-KEY("older adult*")	
	18. TITLE-ABS-KEY("elder*")	
	19. TITLE-ABS-KEY("aging")	
	20. TITLE-ABS-KEY("ageing")	
	21. TITLE-ABS-KEY("elderly")	
	22. TITLE-ABS-KEY("cognitive decline")	
	23. TITLE-ABS-KEY("memory loss")	
	24. TITLE-ABS-KEY("cognitive training")	
	25. TITLE-ABS-KEY("social engagement")	
	26. TITLE-ABS-KEY("intervention")	
	27. TITLE-ABS-KEY("therapy")	
	28. TITLE-ABS-KEY("reminiscence*")	
	29. TITLE-ABS-KEY("cognitive stimulation")	
	30. TITLE-ABS-KEY("caregiver*")	
	31. 1 OR 2 OR 3 OR 4 OR 5 OR 6 OR 7 OR 8 OR 9 OR 10 OR 11 OR 12	
	32. 13 OR 14 OR 15 OR 16 OR 17 OR 18 OR 19 OR 20 OR 21 OR 22 OR 23	
	33. 24 OR 25 OR 26 OR 27 OR 28 OR 29 OR 30	
	34. 31 AND 32 AND 33	
**6. ACM Digital Library**	1. Abstract:(chatbot) OR Title:(chatbot)	**24**
	2. Abstract:("conversational agent") OR Title:("conversational agent")	
	3. Abstract:("dialogue system") OR Title:("dialogue system")	
	4. Abstract:("virtual assistant") OR Title:("virtual assistant")	
	5. Abstract:("conversational AI") OR Title:("conversational AI")	
	6. Abstract:("social robot*") OR Title:("social robot*")	
	7. Abstract:("embodied conversational agent*") OR Title:("embodied conversational agent*")	
	8. Abstract:("large language model*") OR Title:("large language model*")	

	9. Abstract:(LLM*) OR Title:(LLM*)	
	10. Abstract:(ChatGPT) OR Title:(ChatGPT)	
	11. Abstract:("digital companion*") OR Title:("digital companion*")	
	12. Abstract:("voice assistant*") OR Title:("voice assistant*")	
	13. Abstract:(dementia) OR Title:(dementia)	
	14. Abstract:("Alzheimer's disease") OR Title:("Alzheimer's disease")	
	15. Abstract:("cognitive impairment") OR Title:("cognitive impairment")	
	16. Abstract:(MCI) OR Title:(MCI)	
	17. Abstract:("older adult*") OR Title:("older adult*")	
	18. Abstract:(elder*) OR Title:(elder*)	
	19. Abstract:(aging) OR Title:(aging)	
	20. Abstract:(ageing) OR Title:(ageing)	
	21. Abstract:(elderly) OR Title:(elderly)	
	22. Abstract:("cognitive decline") OR Title:("cognitive decline")	
	23. Abstract:("memory loss") OR Title:("memory loss")	
	24. Abstract:("cognitive training") OR Title:("cognitive training")	
	25. Abstract:("social engagement") OR Title:("social engagement")	
	26. Abstract:(intervention) OR Title:(intervention)	
	27. Abstract:(therapy) OR Title:(therapy)	
	28. Abstract:(reminiscence*) OR Title:(reminiscence*)	
	29. Abstract:("cognitive stimulation") OR Title:("cognitive stimulation")	
	30. Abstract:(caregiver*) OR Title:(caregiver*)	
	31. 1 OR 2 OR 3 OR 4 OR 5 OR 6 OR 7 OR 8 OR 9 OR 10 OR 11 OR 12	
	32. 13 OR 14 OR 15 OR 16 OR 17 OR 18 OR 19 OR 20 OR 21 OR 22 OR 23	
	33. 24 OR 25 OR 26 OR 27 OR 28 OR 29 OR 30	
	34. 31 AND 32 AND 33	
**TOTAL before de-duplication**		**3213**
